# A Gene Expression Signature of Acquired Chemoresistance to Cisplatin and Fluorouracil Combination Chemotherapy in Gastric Cancer Patients

**DOI:** 10.1371/journal.pone.0016694

**Published:** 2011-02-18

**Authors:** Hark Kyun Kim, Il Ju Choi, Chan Gyoo Kim, Hee Sung Kim, Akira Oshima, Aleksandra Michalowski, Jeffrey E. Green

**Affiliations:** 1 Laboratory of Cancer Biology and Genetics, National Cancer Institute, Bethesda, Maryland, United States of America; 2 National Cancer Center, Goyang, Republic of Korea; University of Barcelona, Spain

## Abstract

**Background:**

We initiated a prospective trial to identify transcriptional alterations associated with acquired chemotherapy resistance from pre- and post-biopsy samples from the same patient and uncover potential molecular pathways involved in treatment failure to help guide therapeutic alternatives.

**Methodology/Principal Findings:**

A prospective, high-throughput transcriptional profiling study was performed using endoscopic biopsy samples from 123 metastatic gastric cancer patients prior to cisplatin and fluorouracil (CF) combination chemotherapy. 22 patients who initially responded to CF were re-biopsied after they developed resistance to CF. An acquired chemotherapy resistance signature was identified by analyzing the gene expression profiles from the matched pre- and post-CF treated samples.

The acquired resistance signature was able to segregate a separate cohort of 101 newly-diagnosed gastric cancer patients according to the time to progression after CF. Hierarchical clustering using a 633-gene acquired resistance signature (feature selection at *P*<0.01) separated the 101 pretreatment patient samples into two groups with significantly different times to progression (2.5 vs. 4.7 months). This 633-gene signature included the upregulation of *AKT1*, *EIF4B*, and *RPS6* (mTOR pathway), DNA repair and drug metabolism genes, and was enriched for genes overexpressed in embryonic stem cell signatures. A 72-gene acquired resistance signature (a subset of the 633 gene signature also identified in ES cell-related gene sets) was an independent predictor for time to progression (adjusted *P* = 0.011) and survival (adjusted *P* = 0.034) of these 101 patients.

**Conclusion/Significance:**

This signature may offer new insights into identifying new targets and therapies required to overcome the acquired resistance of gastric cancer to CF.

## Introduction

Understanding how tumors evolve on a molecular level to overcome the cytotoxic effects of chemotherapy is a critical step in developing therapeutic approaches that will prevent or overcome chemoresistance. However, due to the difficulties in obtaining serial tumor biopsies from patients at various stages of therapy, the identification of molecular alterations that occur as tumors become resistant to therapy has been a vexing problem. The serial collection of solid tumor samples from the same patients has been extremely difficult in the clinical setting, but gastric cancer provides a unique opportunity for this purpose, since it is often initially responsive to chemotherapy and repeated endoscopies may be performed to monitor tumor response to chemotherapy.

In this study, endoscopic biopsy samples were collected from gastric cancer patients. We identified a gene expression signature for acquired chemoresistance to cisplatin and fluorouracil (CF) combination chemotherapy, by comparing samples collected prior to CF therapy with samples taken from the same patients at the time resistance to CF developed based upon objective clinical progression. Using this approach, we could identify molecular candidates that may possibly lead to development of new targeted therapies for gastric cancer. Importantly, we also found that an acquired chemoresistance signature could identify whether newly diagnosed gastric cancer patients would have a short or more sustained response CF therapy. Since the acquired resistance signature is already highly represented in non-responders and that it seems unlikely that the numerous expression changes occurring on a global level would evolve in a relatively short period of time, our results appear to support the conventional, clonal selection model for tumor progression and acquired chemoresistance [1]. Identifying biomarkers that distinguish cancer patients who will or will not benefit from cytotoxic chemotherapy will greatly improve clinical management. Although studies using high-throughput transcription profiling of pretreatment biopsy samples have attempted to identify such predictors, the performance of these predictors has been mixed [2]. In part, this may be due to the difficulty of identifying robust gene signatures in tumors from populations with large genetic variation. Our data suggests that expression-profiling of posttreatment samples could be a possible alternative approach.

Some studies have suggested that tumors which develop chemoresistance may acquire certain properties inherent to stem cells, and that chemotherapy treatment leads to a concomitant enrichment of cancer stem cells *in vitro* [3]. We further demonstrate that the acquired resistance signature is enriched for genes previously identified in embryonic stem (ES) cell expression signatures, further suggesting that for gastric cancer, chemoresistance arises from selection of pre-existing cells with particular stem cell characteristics.

## Materials and Methods

### Patient accrual and follow-up

This is the part of a prospective trial approved by the Institutional Review Board (IRB) of the National Cancer Center Hospital in Goyang, Korea (NCCNHS01-003). All participants signed an IRB-approved informed consent form. Eligibility for enrollment into the study included the following parameters: 1) age ≥18 years; 2) histologically-confirmed gastric adenocarcinoma; 3) clinically-documented distant metastasis; 4) no previous or concomitant malignancies other than the gastric cancer; 5) no prior history of chemotherapy, either adjuvant or palliative; and 6) adequate function of all major organs. Patients who were lost to follow-up before completing 6 cycles of chemotherapy, except for documented progressive disease, were excluded from the analyses.

Our prospective trial had 2 objectives. The first objective, which is the focus of another paper [4], was to develop a genomic predictor for initial chemotherapy response by correlating the expression profiling data of pretreatment samples with clinical outcome (*intrinsic resistance study*). Sample size of the trial was planned based on this first objective. For the training set, 91 events were estimated to be required at α = 0.001, β = 0.05, τ (standard deviation of log intensity)  =  0.75, and δ (hazard ratio associated with one-unit change of log intensity)  =  2. Hence, 96 pretreatment samples were collected from August 2001 to January 2005 as the training set (for the *intrinsic resistance study*). A second group of 27 eligible patients was enrolled as the array validation cohort between February 2005 and April 2006, which includes 22 patients treated with CF, and 5 patients treated with cisplatin plus oral capecitabine (a fluorouracil pro-drug considered equivalent to fluorouracil; CX). CX therapy was demonstrated to be therapeutically equivalent to the CF regimen for metastatic gastric cancer [5].

The second objective of our prospective trial, which is pursued by analyses presented in this paper, was to identify a gene expression signature for the acquired chemoresistance by comparing pre- and post-treatment samples of the clinical responders (*acquired resistance study*). After an initial endoscopic biopsy, all study patients were prospectively treated and followed-up. Patients were treated with cisplatin (60 mg/m^2^, D1) in combination with either fluorouracil (1 g/m^2^ for 5 days; n = 118) or capecitabine (Xeloda; Roche; 1,250 mg/m^2^ BID for 2 weeks; n = 5)^5^ every 3 weeks. Chemotherapy doses were reduced depending upon toxicities and the patient's performance status. Specific dose modification schemes for the subsequent treatment cycle were at the discretion of the attending oncologist. The treatment schedule for fluorouracil could be shortened at the discretion of the oncologist from 5 to 3 days for elderly patients (≥70 years) or patients with a poor performance status (Eastern Cooperative Oncology Group (ECOG) performance status ≥2). Abdominal spiral computed tomography (CT) scans were performed for all patients every 3 cycles of chemotherapy (*i.e.*, 9 weeks). Objective response was documented for patients with measurable disease according to World Health Organization (WHO) criteria [6]. A partial response (PR) was defined as more than a 50% decrease in the sum of the products of the 2 largest perpendicular diameters of measurable lesions for at least 4 weeks, but a confirmation CT was not routinely performed 4 weeks after the initial documentation of PR.

There were 38 patients with PR among 96 training set (of the *intrinsic resistance study*). Patients with PR underwent a follow-up biopsy at the time disease progression was observed (*i.e.*, progressive disease according to the WHO criteria), referred to as the “chemoresistant state”. Adequate biopsy samples from tumors in a chemoresistant state were available from 22 patients with PR (57.9%). Chemoresistance state biopsy samples of the other 16 patients (42.1%) could not be profiled due to either inadequate RNA quantity/quality or patients' refusal. There was no difference in age, sex, histological type, time to progression (TTP), and overall survival between 22 re-biopsied patients and the other 16 patients who had PR but were not re-biopsied. Samples were collected at least 2 weeks after the last dose of the fluorouracil *and* before second-line chemotherapy was started, in order to minimize any acute drug effects on expression profile.

Two pieces of grossly-normal gastric mucosa tissue samples were also collected from antrum of 21 healthy volunteers (Table S1).

### Identification of an acquired resistance signature to CF

Endoscopic biopsies were performed to obtain the fresh tissue. Five to ten pieces of fresh tumor tissues were obtained from non-necrotic portion of tumor using large cup biopsy forceps of 7.3 mm diameter (Olympus FB-24K-1, Olympus, Tokyo, Japan). Then obtained fresh tissues were frozen in liquid nitrogen within 15 min of the first biopsy harvest. Tissue samples containing at least 50% tumor cells were processed for RNA as previously described [7]. One microgram of total RNA was amplified and hybridized to an HG-U133A cartridge array, according to the manufacturer's protocol (Affymetrix, Santa Clara, CA). All expression microarray data is available at the Gene Expression Omnibus (GEO) Database (accession number GSE14210, http://www.ncbi.nlm.nih.gov/geo) [CURRENTLY, REVIEWER ACCESS ONLY: http://www.ncbi.nlm.nih.gov/geo/query/acc.cgi?token=rtgnlocqoqeiwtw&acc=GSE14210]. Gene expression microarray data were normalized by Robust Multichip Average (RMA) using R2.6. Pre- and post-CF expression data from 22 rebiopsied responders were normalized independently from the expression data from a separate group of 101 non-rebiopsied patients. Microarray data were analyzed using BRB ArrayTools (version 3.6, National Cancer Institute, http://linus.nci.nih.gov/BRB-ArrayTools.html) [8].

Gene expression changes that distinguished the initial transcriptional status of tumors from gene expression patterns when tumors became chemoresistant were determined for the 22 patients with documented initial response (PR) to CF therapy. Matched microarray data was compared between the samples obtained prior to CF treatment and samples collected after resistance to therapy developed. These data were analyzed using the class comparison algorithm of BRB-ArrayTools (random variance model), which computes a paired *t*-test for each gene using the RMA-summarized log-intensities for Affymetrix U133A arrays. Genes differentially expressed between these 22 paired samples defined the acquired resistance signature. At feature selection *P*-value cutoffs of 0.05 and 0.01, a permutation P value was calculated, which is the proportion of random permutations that identify a similar number of significant genes that are found when comparing the true class labels.

Time to progression was plotted using the Kaplan-Meier method. A log-rank test was used to determine differences between survival curves. Wald's test was used to assess the statistical significance of the Cox hazard ratio. Multivariable regression analyses were performed using a Cox proportional hazard model. All these analyses were performed using SPSS (version 15.0; SPSS, Inc., Chicago, IL). Multivariable ordinal logistic regression analysis was performed using to SAS (version 9.1.3, SAS, Cary, NC), to evaluate the association between the 72-gene predictive index and radiographic response.

### Transcription factor analysis

Transcription factor analyses were performed to look for the enrichment of transcription factor targets in the genes comprising the acquired resistance signature (BRB-ArrayTools). All genes queried in this analysis algorithm have been catalogued to transcription factor responsive categories based upon experimentally-verified transcription factor responsiveness. Transcription factor-binding curation information in the Transcriptional Regulatory Element Database (TRED) [9] was used to eliminate targets without any experimental verification.

### Analysis of public DNA microarray data from surgically treated gastric cancer patients

Publicly accessible microarray data for surgically-treated gastric patients generated by the Stanford Functional Genomics Facility were also obtained from the NCBI GEO database (GSE4007) and included about 30,300 genes common to these datasets. The microarray data were generated and normalized as described in Leung *et al* [10]. Batch effects in gene expression were removed with probe-wise mean centering and missing data were imputed with the nearest neighbor averaging method [11]. The array cDNA clones were annotated using SOURCE (Stanford Microarray Database) and the Entrez GeneID was used as the mapping identifier for the Affymetrix HG-U133A array.

### Gene set comparison analyses

The gene set comparison tool analyzes user-defined gene sets for differential expression among pre-defined classes of a source dataset. For each source dataset, a *P*-value is computed for each gene to correlate the expression level *vs.* survival time using a proportional hazards model (or for the differential expression between pre-defined classes, depending on the nature of the phenotype), generating a ranked gene list of a given BRB-ArrayTools project. For a set of *N* genes, the LS statistic is defined as the mean negative natural logarithm of the *P*-values of the appropriate single gene univariate tests [12]. A summary statistic is computed that summarizes these *P* values over the user-defined gene set; the summary statistic is average log(*P*) for the LS summary of how the *P* values differ from a uniform distribution for LS [12]. The summary statistic is related to the distribution of the summary statistics for random samples of *N* genes, sampled from those represented on the array. Here *N* is the number of genes in the user-defined gene set. 100,000 random gene sets were sampled to compute this distribution. The LS *P* value is the proportion of random sets of *N* genes with smaller average summary statistics than the LS summaries computed for the real data. This approach is used for a variety of types of correlations between gene expression levels and phenotype. The nature of the phenotype (for instance, survival time or binary indicators) determines the manner in which the gene specific *P* values are computed. An LS *P* value less than 0.005 is considered significant.

### Identification of a gastric cancer-specific signature and a gastric cancer differentiation signature

Total RNA was isolated from frozen endoscopic biopsy samples of the antral mucosa collected from 21 healthy volunteers and analyzed by microarray as previously described. In order to identify the gastric cancer-specific signature, we compared the expression data from the 21 normal samples with 101 samples from patients prior to chemotherapy samples (excluding 22 rebiopsied patients used to develop the acquired resistance signature) using class comparison algorithms of BRB-ArrayTools.

Of the 101 patients, 41 patients had Lauren's intestinal histological type of primary tumors and 60 had the diffuse type. Mixed-type tumors were categorized together with the diffuse type. A differentiation signature was identified by comparing the gene expression data from the 41 intestinal type samples with 60 diffuse type samples using class comparison algorithms of BRB-ArrayTools.

### Generation of ES cell signatures from published data

To generate a user-defined gene set for our gene comparison analyses, we adopted several gene lists from the published work of Ben-Porath *et al* [13], in which several gene sets associated with ES cell identity were compiled for gene set comparison analyses. An “*ES expression set*” was previously defined by Ben-Porath *et al* [13] as genes over-expressed in human ES cells in at least 5 out of 20 profiling studies [14]. This ES expression set was then amended [13] so that genes in the “proliferation” Gene Ontology and the proliferation cluster of breast cancer [13], [15] were excluded and referred to as the *ES set without proliferation genes*. Lists of target genes for MYC [16], SOX2 [17], OCT4 [17], NANOG [17], SUZ12 [18], EED [18], and H3K27 [18], which are key transcription factors in stem cells, were also adopted from Ben-Porath [13]. These genes were originally identified by chromatin immunoprecipitation array studies [16]–[18]. For our gene set comparison analyses, Entrez IDs [13] of target genes were mapped to probe set IDs on the HG-U133A array (www.NetAffx.com).

### Identification of a 72-gene predictive index

Among the 468 genes upregulated at the chemoresistant state (*P*<0.01), 72 unique genes were members of at least one of 4 published *ES cell-related gene sets* (“*ES set without proliferation genes*” [13], [15], the experimentally-validated MYC transcription factor target gene set (TRED MYC_T00140) [9], and target genes of MYC and SOX2 identified by a chromatin immunoprecipitation array study [16], [17]). A genomic predictor (referred to as the “72-gene predictive index”) was constructed by calculating the weighted linear combination of log signal values of these 72 unique genes overlapping between the acquired resistance signature and “*ES cell-related gene sets*”. The univariate *t*-statistics for comparing the classes (acquired chemoresistant *vs.* pretreatment states) were used as the weights. BRB-ArrayTools (the class prediction) was used to calculate the *t*-value of each gene. The predictive power of the 72-gene predictive index was tested for time to progression and survival using the Cox proportional hazards model.

## Results

### Identification of an acquired resistance signature to CF

Twenty-two patients who demonstrated a clinical response (PR) to CF therapy were biopsied prior to the initiation of therapy and subsequently following progression of disease after chemotherapy. Pre- and post-CF samples were not significantly different in the tumor cell percentage and measures of microarray data quality control (Table 1 and Table S2). Median interval between the 2 biopsies was 8.7 months (interquartile range, 6.4–12.6). Since the permutation *P* values were consistently less than 0.05 at *P* cutoffs for feature selection of 0.01 and 0.05 (permutation *P* values, 0.012 and 0.006, respectively), this demonstrates that gene expression is significantly different between the chemoresistant and pretreatment states. Genes differentially expressed between the pretreatment state of 22 tumors that proved initially responsive to CF chemotherapy and tumors from the same patients after having evolved into an acquired chemoresistant state were identified as the “acquired resistance signatures”. 2,446 genes were identified in the acquired resistance signature with a feature selection of *P*<0.05, whereas 633 genes were identified using a feature selection of *P*<0.01. The most highly represented functional category in the acquired resistance signature was *Protein Synthesis* (Table S3; Ingenuity Pathway Analysis [www.ingenuity.com]), which includes *AKT1*, ribosomal subunit mRNAs (*RPS6, RPL13, RPL14, RPL15, RPL18, RPL29, RPL3, RPL30, RPL4, RPS11, RPS19, RPS9*), and eukaryotic translation initiation factors (*EIF4B EIF3D, EIF3E, EIF3F, EIF3H*). Akt/mTOR and Ras–MAPK signaling modules are two most-studied pathways that exhibit a paramount effect on translational regulation [19]. Given the concurrent upregulation of these key components of this pathway (*AKT1* (*P* = 0.0012), *EIF4B* (*P* = 0.0089), and *RPS6* (*P* = 0.0009)), the PI3K/Akt/mTOR signal transduction pathway is presumed to be activated in the acquired resistance state (Figure S1). AKT1 has been linked to *in vitro* cisplatin resistance [20]–[22]. mTOR inhibition has also been known to reverse *in vitro* acquired resistance to endocrine therapy and EGFR inhibitors of breast and lung cancers, respectively [23], [24]. Since *ERBB2* is also upregulated in the acquired resistance signature (*P* = 0.0065), *ERBB2* may play a role in the upregulation of *Protein Synthesis*-related genes, through activation of the mTOR pathway [25].

**Table 1:** Clinicopathological Characteristics of Patient Subgroups Used for This Analysis.

	Rebiopsied	Non-rebiopsied
	(N = 22)	(N = 101)
***Baseline clinicopathological characteristic***		
**Age (years)**		
Median	58	58
Interquartile range	(52–63)	(52–64)
**Sex – no. (%)**		
Male	20 (90.9%)	76 (72.2%)
Female	2 (9.1%)	25 (27.8%)
**Performance status (PS) – no. (%)**		
ECOG^1^ PS 0 or 1	22 (100%)	94 (93.1%)
ECOG PS 2 or 3	0 (0.0%)	7 (6.9%)
**Histological type – no. (%)**		
Lauren's intestinal	8 (36.4%)	41(40.6%)
Lauren's diffuse	14 (63.6%)	60 (59.4%)
**Location of primary lesion – no. (%)**		
Upper 1/3	1 (0.5%)	15 (14.9%)
Middle 1/3	7 (31.8%)	31 (30.7%)
Lower 1/3	13 (59.1%)	51 (50.5%)
Entire stomach	1 (0.5%)	4 (4.1%)
Distant metastasis – no. (%)	22 (100%)	101 (100%)
**Tumor cell percentage in sample – (%)**		
Median	60	60
Interquartile range	(50–78)	(50–70)
***Treatment and outcome***		
**Chemotherapy regimen – no. (%)**		
Cisplatin/Fluorouracil	22 (100%)	96 (95.0%)
Cisplatin/Capecitabine	0 (0%)	5 (5.0%)
**Relative dose intensity - %**		
Median	73	80
Interquartile range	68–82	74–90
**Number of chemotherapy cycles**		
Median	10	4
Interquartile range	(9–15)	(3–8)
**Radiographic response (WHO criteria) – no. (%)**		
PR^2^	22 (100.0%)	28 (31.8%)
SD^3^	0	28 (31.8%)
PD^4^	0	32 (36.4%)
Nonmeasurable disease	0	13
**Second-line chemotherapy – no. (%)**	19 (86.4%)	69 (68.3%)
**Median follow-up for survivors – mo.**		35.5
**Time to progression – mo.**		
Median	8	3.9
Interquartile range	(5.6–12.5)	(2.3–8.6)
**Overall survival – mo.**		
Median	11.9	7.9
Interquartile range	(11.3–21.1)	(5.6–16.8)

1 Eastern Cooperative Oncology Group,

2 Partial Response,

3 Stable Disease, and,

4 Progressive Disease.

Transcription factor gene set comparison analysis indicated that the acquired resistance signature is enriched with targets of multiple transcription factors, including a MYC target gene set (TRED MYC_T00140) [9] (Table S4). This is consistent with a microarray data in the literature that the majority of genes responsive to Myc overexpression are involved in macromolecular synthesis, protein turnover, and metabolism, including 30 ribosomal protein genes [26].

### The acquired resistance signature segregates patients according to the time to disease progression following CF therapy, but is not prognostic in gastric cancer patients treated only by surgery

**Figure 1:**
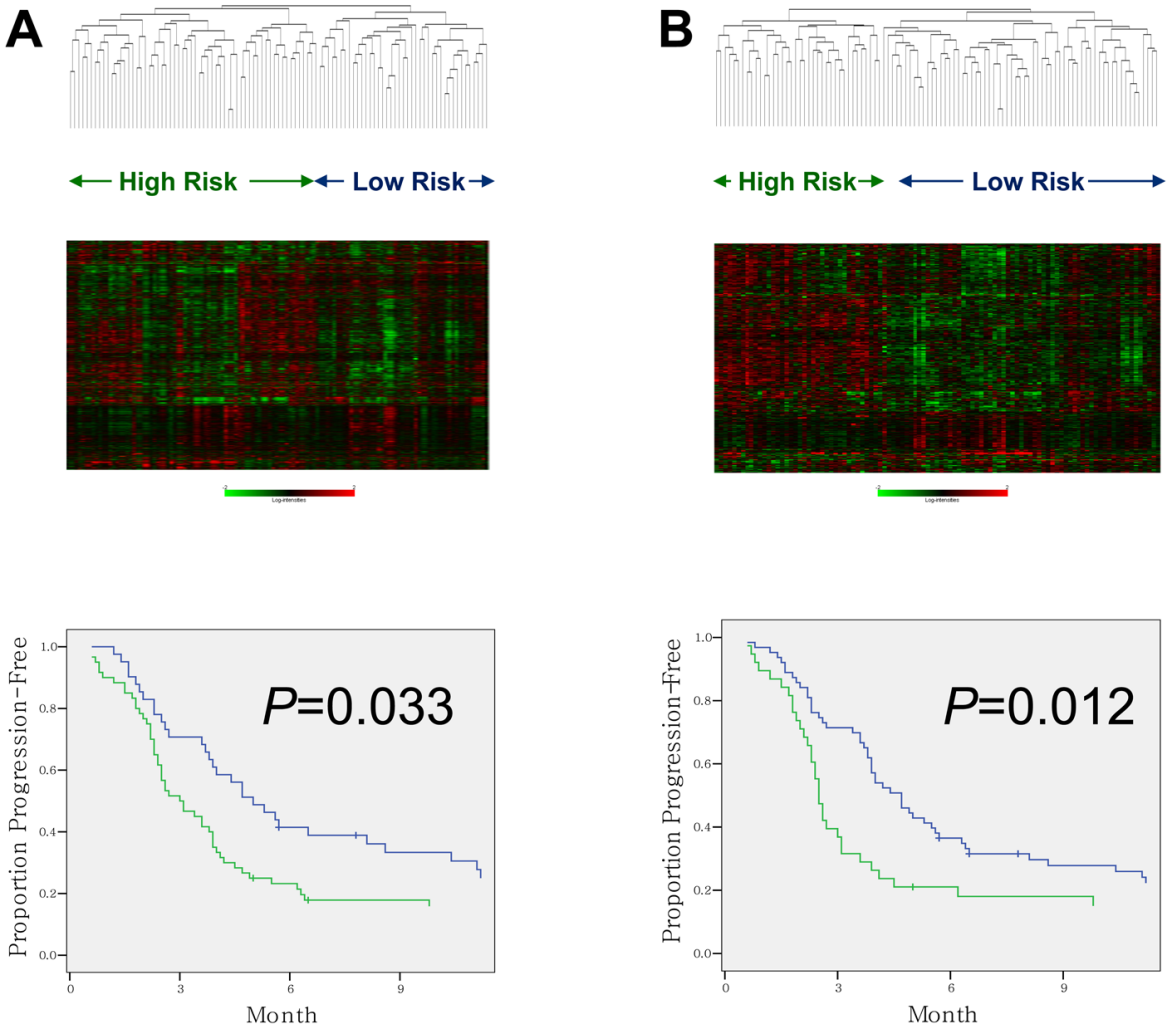
Hieraching clustering analyses of pretreatment samples using acquired resistance signatures. Hierarchical clustering dendrograms of pretreatment samples from a separate set of 101 gastric cancer patients, using genes differentially expressed between the pretreatment- and chemoresistant-states of 22 rebiopsied responders at various *P* cutoffs for feature selection. Kaplan-Meier plots for the time to progression (TTP) calculated for each of the two major clusters generated by each dendrogram are shown below. (A) Hierarchical clustering of 101 pretreatment samples using the 2,446–gene acquired resistance signature (*P* for feature selection<0.05). Heatmap generated using a log_2_-pseudocolor image with gene centering. Kaplan-Meier plots for TTP calculated for each of the two major clusters generated are shown below. Patients in high risk cluster (n = 60, high expression of the genes upregulated at chemoresistant state (*upper*) had a significantly shorter TTP than patients in low risk cluster (n = 41, low expression) (3.0 *vs*. 5.0 months; *P* = 0.033). (B) Hierarchical clustering of the same 101 gastric cancer samples using the 633–gene acquired resistance signature (*P* for feature selection<0.01). Patients in high risk cluster (n = 38, high expression of genes upregulated at chemoresistant state (*upper*) had a significantly shorter TTP than patients in low risk cluster (n = 63, low expression) (2.5 *vs*. 4.7 months; *P* = 0.012).

We wished to determine whether expression of the acquired resistance signature in gastric cancer tumors at initial diagnosis was predictive of response to CF therapy. Expression of the acquired resistance signature in a separate group of 101 non-rebiopsied gastric cancer patients was determined and related to the clinical outcome of the patients according to which major hierarchical cluster the patients were grouped. In patients without lesions initially measurable by diagnostic imaging, time to progression (TTP) was measured from the initiation of CF therapy to the time when a change in therapy was required due to unequivocal disease progression. Hierarchical clustering of the 101 pretreatment samples was performed using the 2,446-gene acquired resistance signature. Outcome as measured by TTP was significantly different between patients in each of the two primary clusters. Patients in the cluster with increased expression of the genes upregulated in the chemoresistant state had a significantly shorter TTP than patients with lower expression of these genes (Log-rank *P* value = 0.033) (Figure 1A). In order to further evaluate the association of these 2,446 genes with TTP of 101 patients, we also performed a survival risk prediction analysis of BRB-ArrayTools, in which the entire 10-fold cross-validation process was repeated using 2,446 genes and a log-rank statistic for TTP between 2 predicted risk groups was obtained for each random dataset with TTP data shuffled among 101 patients [8], [27]. The permutation *P* value for testing the null hypothesis that there is no relation between 2,446 genes and TTP, which is the tail area of this null distribution beyond the log-rank value obtained for the real data, was estimated 0.06, suggesting a borderline significance of the association. Patients in the cluster with increased expression of 468 genes upregulated in the chemoresistant state at *P*<0.01 also had a significantly shorter TTP than patients with lower expression of these genes (Log-rank *P* value = 0.012) (Figure 1B). These results suggest that the acquired resistance signature reflects real molecular profile of chemoresistant clones, not nonspecific drug effects.

We also wished to further address whether these acquired resistance signatures were predictive of CF response or represented a general prognostic signature that could predict survival of 88 gastric cancer patients who were treated by surgery alone and not by chemotherapy^10^. Neither of the two acquired resistance signatures (2,446 or 633 genes) was predictive of survival in the surgically treated gastric cancer patients using the same hierarchical clustering method as above (Log-rank *P* values, 0.84 and 0.41, respectively). Thus, the acquired resistance signature is predictive of patient response to CF and not just prognostic for gastric cancer patients in general.

### The acquired resistance signature shares many features with the intrinsic resistance signature, but not with a gastric cancer-specific signature or a gastric cancer differentiation signature

**Figure 2:**
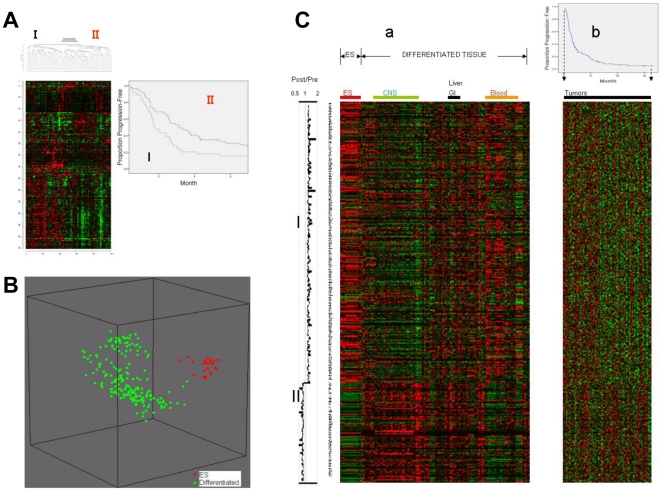
Acquired resistance signature and stem cell signature. (A) Hierarchical clustering of 101 pretreatment patient samples using the “ES set without proliferation genes signature”. Kaplan-Meier plots for time to progression (TTP) of patients in each cluster generated are shown on the right side. Patients in high risk cluster (I) (n = 44, high expression of “ES set without proliferation genes”) had a significantly shorter TTP than patients in the low risk cluster (II) (n = 57, low expression) (2.7 *vs.* 4.7 months; Log-rank *P* value = 0.014). (B) Principal component analysis plot using a published U133A microarray meta-analysis dataset [14] containing 24 human ES cell samples (*shown in red*) and 193 various fetal and adult differentiated tissue samples (*shown in green*) using the 633-gene acquired resistance signature (feature selection *P*<0.01). Each sphere represents a single sample. Samples whose expression profiles of 633 genes are similar are shown close together. (C) *a*. Expression of the 633-gene acquired resistance signature using the same published meta-analysis microarray data^14^ as in (B). Heatmap generated using a log_2_-pseudocolor image with gene centering. Red and green colors represent high and low gene expression levels, respectively. Genes upregulated at the chemoresistant state of our study patients (post/pre>1, I) show coordinated overexpression in ES cells (*left*), while genes downregulated at the chemoresistant state (post/pre<1, II) show coordinated overexpression in differentiated tissue samples (*right*). *b*. Expression of the same 633-gene acquired resistance signature in 101 pretreatment samples collected from a separate set of gastric cancer patients. Each row represents each patient, sorted according to the increasing order of TTP from left to right, as matched with Kaplan-Meier curves for TTP of 101 patients (*top right*). Genes upregulated at the chemoresistant state of our study patients (I) show the concordant overexpression in patients with shorter TTP (*left*), while genes downregulated at the chemoresistant state (II) show the concordant overexpression in patients with longer TTP (*right*).

These acquired resistance signatures were then compared with the intrinsic drug resistance signature of a separate group of 101 non-rebiopsied patients, using gene set comparison analysis of BRB-ArrayTools [12]. Briefly, this algorithm computed a *P*-value for each of 2,446 genes to correlate the expression level *vs.* TTP of these 101 patients using a proportional hazards model. Then it computed mean negative natural logarithm of the *P*-values of the single gene univariate tests (LS statistic of this set of 2,446 genes) and the proportion of random sets of 2,446 genes with smaller average summary statistics than the LS summaries computed for the real data (LS *P* value). The same analysis was repeated for 633 genes selected at *P*<0.01. Consistent with results of the hierarchical clustering analyses, the acquired resistance signatures were found to be highly enriched in the “intrinsic resistance signature” of a separate group of 101 CF-treated patients. LS re-sampling *P* values were <10^−5^ for both user-defined gene sets selected with different cutoffs to define the acquired resistance signature (*i.e.*, for 2,446 and 633 genes). Genes overlapping between acquired and intrinsic resistance signatures are listed in Table 2. Figure 2C *b* graphically displays that 468 genes upregulated at the chemoresistant state of 22 rebiopsied patients (*P*<0.01) show the concordant overexpression in non-rebiopsied patients with shorter TTP, while 165 genes downregulated at the chemoresistant state show the concordant overexpression in patients with longer TTP.

**Table 2:** Genes Which Belong to the Acquired Resistance Signature (selected at *P*<0.05) *and* Were Correlated with TTP of 101 Non-rebiopsied Patients at *P*<0.05.

Upregulated at												Downregulated at	
chemoresistant state^1^												chemoresistant state^1^	
Gene	HR^2^	Gene	HR	Gene	HR	Gene	HR	Gene	HR	Gene	HR	Gene	HR
DEXI	3.1	RPS20	4.4	INTS8	1.7	PA2G4	1.7	MINA	1.5	SLC29A2	1.5	CYorf14	0.6
TRAP1	1.9	TIMM10	1.9	COPS5	1.7	PSMD4	1.8	EIF4B	2.1	RPL3	2.4	SYTL2	0.7
HIST3H2A	1.7	LGTN	2.0	RPS19	2.4	C10orf2	1.5	PSMD4	1.6	FAU	1.9	PTPRD	2.3
RPL13P12	3.4	RPS19	2.5	SQLE	1.3	E2F5	1.4	ATP5G1	1.6	EXOSC4	1.4	SEC24D	0.6
RPL13	5.4	RPL13	3.0	POLR2G	1.8	SCRN3	0.5	APRT	1.5	DNAJB12	1.7	TLE4	0.4
RPS20	3.8	MPHOSPH10	2.2	MEST	1.3	EEF1B2	1.8	EXOSC5	1.4	RPS9	1.9	214101_s_at	1.6
ATIC	1.9	AOF2	1.8	SNRPD2	1.6	NAT10	1.6	OSBPL1A	0.8	CCNB1IP1	1.3	CASP1	0.7
EIF3H	2.7	NHP2L1	1.9	RPS18	3.1	CDK4	1.5	NOL7	1.5	EIF3L	1.8	KLHL2	0.5
NENF	2.6	CENPN	1.4	RRP12	1.7	CDK5RAP1	1.6	KLHDC3	1.6	MRPL11	1.4	SAT1	0.6
RPL8	2.3	PHB2	1.9	PKN2	0.8	SMYD5	1.7	SPTLC2	1.6	METRN	1.5	207799_x_at	2.4
RBMX	2.5	RNASEH2A	1.5	PHB	1.5	RANBP1	1.4	RPS5	1.8	DDB1	1.8	IL15	0.7
PRMT5	1.8	ADSL	1.6	PTK2B	0.6	H2AFY	0.7	TNFRSF11A	0.7	LONP1	1.6	ARNTL	0.5
PCBD1	1.8	COX4NB	1.8	MRPL13	1.6	RPS3	2.2	EIF2B4	1.6	RPL12	2.1	JAK2	0.7
FDPS	2.2	FXC1	2.3	DGUOK	2.2	VKORC1	1.6	POLG2	1.6	RPL3	2.7	GATA3	1.3
DAP3	2.3	ENY2	1.8	STOML2	1.5	DDX19A	1.8	EIF3C	1.6	GLG1	1.6	ANTXR1	2.3
LAS1L	1.9	NBAS	2.5	EEF1G	2.6	PFDN2	1.5	TRMT12	1.4	DDAH2	1.3	SEC24D	0.5
C15orf44	2.9	TBRG4	1.7	ELP3	1.9	IMP4	1.5	NRTN	2.2	CPNE3	1.5	EIF1AY	0.7
RAN	1.8	EIF4EBP1	1.4	RPL32	3.0	GCSH	1.5	TFDP2	1.4	ICT1	1.4	STX12	0.6
TRMT1	1.7	TBCB	1.6	HMBS	1.8	NELF	1.5	C14orf1	1.5	VARS	1.3	KCNJ15	0.8
UTP14A	1.6	POLR2C	2.1	MOSC1	1.6	LMNB2	1.6	RPL38	2.3	ITPA	1.8	LIMS1	1.5
SNRPE	1.9	Magmas	1.8	HTRA2	1.9	DDX28	1.5	RPL14	1.7	GP2	0.9	CCRL2	0.7
RPL13	3.8	RPP40	1.5	M10098_5_at	0.9	SSSCA1	1.5	ZNF259	1.6	SF3B2	1.9	CYP19A1	1.5
SNRPB	1.7	PCCB	1.7	NDUFA13	1.7	GNB2L1	1.8	NLE1	1.4	SGSM3	0.7	GATA3	1.3
PUS1	2.0	GTPBP4	1.6	DDX28	2.0	FOXJ1	1.6	CSE1L	1.3	RPL12	2.2	SPG20	0.7
RUVBL1	1.5	TH1L	1.5	NME1	1.4	M6PR	1.6	RPL15	2.0	FUS	1.5	JTV1	1.6
PUF60	1.9	UMPS	1.8	INTS5	1.9	MCM3	1.4	BANF1	1.4	YY1	1.5	AMPD1	0.7
RPL13	4.1	RPS15	2.2	CSE1L	1.4	MYLIP	1.5	C17orf90	1.7	EIF3CL	1.6	KCNJ15	0.7
NUP93	1.8	TRMT1	1.7	ADSL	1.6	CNPY2	1.6	MED18	0.5	CHCHD8	1.6	AZIN1	1.7
PPIE	2.1	IPO5	1.5	CSTF2	1.9	EEF1G	2.3	FLAD1	1.5	PSMB7	1.4	KLF10	1.5
RPL36A	2.4	CKAP5	1.8	PTRH2	1.6	DHX30	1.6	MAN1B1	2.0	RPL4	1.6	DDN	1.8
EIF3F	2.4	EIF3E	1.9	RPL24	2.2	PSMB4	1.7	FTSJ2	1.6	CDCA4	1.4	DDX3Y	0.7
CCT7	2.0	PES1	1.9	DDX31	1.7	ALDH7A1	1.5	DNPEP	1.6	PAAF1	1.3	SEMA3F	0.7
HSPC152	2.1	SNRPC	2.2	MMS19	1.7	TNFSF13	0.8	GPR172A	1.4	POLR2H	1.6	NR3C1	0.7
ERI3	1.9	MRPL15	1.6	WDR74	1.6	C16orf58	2.0	LOC552889	0.7	KATNB1	1.5	KLF2	0.8
TTC27	1.6	NHP2	1.7	SQLE	1.4	DDX49	1.8	CLNS1A	1.4	CHST5	1.5	LOC100130354	2.9
PMPCA	2.1	SMARCC1	1.5	POLR2I	1.5	MTX1	1.5	MCM7	1.3	C6orf48	1.3	ALK	2.7
EIF3D	2.2	RPP21	1.9	RDBP	1.5	ZNF593	1.4	MDN1	1.5	FLAD1	1.5	HS3ST3A1	1.4
RUVBL2	1.7	RPL6	2.6	NOP56	1.4	ATP1A1	0.7	CDC34	1.5	LY6E	1.2	SEC24A	0.7
TDP1	1.8	APEX1	1.6	TJP3	0.8	NFATC2IP	0.7	TEX264	1.7	NUDC	1.4	PTPRR	0.8
MRPL24	1.9	TMEM147	1.6	RPS21	1.8	MRPS28	1.4	APRT	1.5	TRAPPC2L	1.5	EDA	1.6
KARS	2.2	NSUN5	1.7	EHF	0.8	CPSF1	1.5	OPA3	0.6	AGFG1	0.7	EIF2C4	0.5
PPIE	1.9	CNPY3	1.6	NOL7	1.6	MACROD1	1.4	MRPS7	1.5	RPLP0P6	1.7	INHBC	1.4
LIG3	2.1	CIAPIN1	2.0	TUFM	1.7	GADD45GIP1	1.4	TUSC4	1.8	RPL29	1.6	SEZ6L	1.7
RPL31	3.3	HMGA1	1.4	LANCL2	1.5	GP2	0.8	GPR175	1.7			EIF1AY	0.9
RPL10A	2.3	BCAT2	1.6	ANAPC5	1.6	POLR1C	1.5	GPSN2	1.4			SLC6A6	0.5
RPL30	3.2	RPL18	2.2	SMARCA4	1.6	RPL12	2.4	TTC38	0.7				
RPL4	2.2	POP5	1.8	RNF8	1.8	PRPF3	1.6	FAM86B1	1.4				
C8orf55	1.6	CSNK1A1	0.6	EMG1	1.5	EEF1G	2.4	LSM4	1.4				
RPL4	2.2	MED20	1.6	PACSIN3	1.7	KAT2A	1.6	THG1L	1.6				
DDX1	1.7	CCT3	1.6	THAP11	1.9	TSFM	1.7	MRPS2	1.4				

1 Sorted according to the increasing order of *P* value for TTP correlation.

2 The ratio of hazards of disease progression of 101 patients for a two-fold change in the gene expression level.

Using similar gene set comparison analyses, acquired resistance signatures were then compared with “*gastric cancer-specific signature*” and *“gastric cancer differentiation signature*” of these 101 patients. To compare the acquired resistance signature with “*gastric cancer-specific signature*”, the LS statistic of 2,446 genes in the acquired resistance signature was estimated by computing a mean negative natural logarithm of the *P*-values of the single gene univariate tests for differential expression of each of 2,446 genes between 101 gastric cancer patients and 21 healthy volunteers. No significant overlap in gene expression was observed comparing the acquired resistance signature to a “*gastric cancer-specific signature*” (LS *P* value = 0.96; Table S5). Similarly, there was no significant overlap between the 2,446-gene acquired resistance signature and a*“gastric cancer differentiation signature*” that we identified through the comparison of gene expression between Lauren's intestinal- (n = 41) *vs.* diffuse-type (n = 60) tumors, either (LS *P* value = 0.024; Table S5). These results further suggest that the acquired resistance signature represents a set of genes dysregulated in association with chemoresistance, and not cancer in general.

### The acquired resistance signature shares features with stem cell signatures

Given that complex regulatory networks in stem cells can be best detected by expression analysis of many genes, we performed several gene set comparison analyses comparing our acquired resistance signatures with published ES cell signatures as reported by Ben-Porath *et al* ^13^ (Table S6). We hypothesized that comparing the acquired resistance signature with ES cell signatures would be informative, since it has been suggested that cancer progenitor cells possess stem cell-like traits [28].

Our acquired resistance signature, unlike the *gastric cancer-specific signature* or the *gastric cancer differentiation signature*, was found to be highly enriched for genes contained in the *ES expression set* (defined as genes over-expressed in at least 5/20 human ES cells profiling studies [14]) (LS *P* value = 3.0×10^−3^; Tables S5 and S6). Since ES cells are highly proliferative *in vitro* while stem cells are generally quiescent *in vivo*, the “*ES expression set*” was modified^13^ to exclude genes listed in the “proliferation” category of Gene Ontology and the proliferation cluster of breast cancer^15^. This amended ES expression set (designated the *ES set without proliferation genes*) could also segregate the 101 pretreatment tumor samples according to time to progression. Patients in the high risk cluster (n = 44, high expression of “*ES set without proliferation genes*”) had a significantly shorter TTP than patients in the low risk cluster (n = 57, low expression) (2.7 *vs*. 4.7 months; Log-rank *P* value = 0.014) (Figure 2A). Notably, the overlap between “*ES set without proliferation genes*” and our acquired resistance signature was still statistically significant (LS *P* value = 4.0×10^−3^). Among individual stem cell transcription factor target gene sets, target genes of MYC [16] and SOX2 [17], which are known to be overexpressed in ES cells [13], were enriched in the acquired resistance signature (LS *P* values, 1.0×10^−5^ and 4.3×10^−4^, respectively), while target genes of NANOG or OCT4 were not (Table S6). Figure 2C *a* depicts the graphic representation of the coordinated over- or under-expression of genes upregulated in the chemoresistant state in published microarray data [14] for ES- and differentiated-cells, respectively.

**Figure 3:**
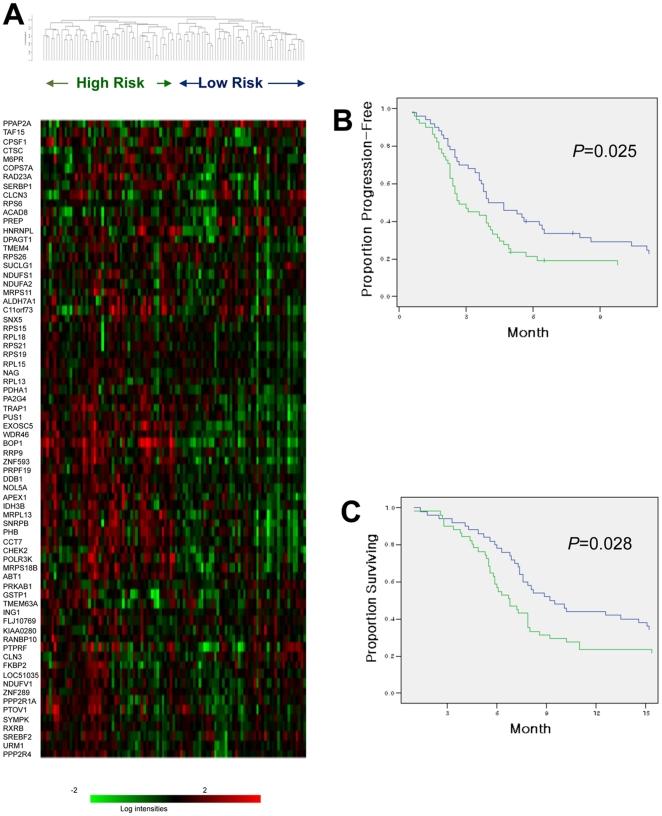
Hierarchical clustering analyses of pretreatment samples using the 72 genes. (A) Hierarchical clustering of the 101 gastric cancer samples using the 72 genes that are upregulated at chemoresistant state (*P*<0.01) and belong to “ES cell-related gene sets”. (B) Patients in high risk cluster according to (A) (n = 51, high expression of 72 genes) had a significantly shorter time to progression (TTP) than patients in low risk cluster (n = 50, low expression) (2.7 *vs.* 4.0 months; *P* = 0.025). (C) Patients in high risk cluster according to (A) (n = 51, high expression of 72 genes) had a significantly shorter survival than patients in low risk cluster (n = 50, low expression) (6.8 *vs.* 9.2 months; *P* = 0.028).

We, therefore, wished to test a hypothesis that ES cell signatures might actually represent a core set of genes associated with in the acquired resistance. We focused on gene sets representing ES cell signatures that significantly overlap with the acquired resistance signature - *i.e.*, “*ES set without proliferation genes*” and target genes of MYC and SOX2 - (designated *ES cell-related gene sets*). Since these gene sets are known to be overexpressed in ES cells^13^, we extracted 72 unique genes, which belong to these “*ES cell-related gene sets*” and were upregulated in the chemoresistant state at *P*<0.01 (designated the *72-gene acquired resistance signature*; Table 3), from 633 genes in the acquired resistance (*P*<0.01). Using this “*72-gene acquired resistance signature*”, hierarchical clustering was performed using a separate set of 101 pretreatment gastric cancer samples from patients who were subsequently treated with CF and were not re-biopsied. This generated two main clusters (Figure 3A) where patients in the high expression cluster exhibited more rapid disease progression and poorer survival than patients in the cluster with lower expression (Log-rank *P* values, 0.025 and 0.028) (Figures 3B and 3C). The multivariable regression analyses demonstrated that the 72-gene predictive index, as a continuous variable, is an independent predictor for time to progression, overall survival, and radiographic response, after adjusted for age, sex, and performance status (Table 4). Prominent among these 72 genes are anti-apoptotic genes (*TRAP1* [29], *CLD3* [30]) and DNA repair (*RAD23A* [31], *DDB1* [32]) and detoxifying enzymes (*GSTP1* [33], [34]), which are associated with chemotherapy resistance *in vitro*. Notably, 50 out of these 72 genes are MYC target genes [26]. MYC is sufficient to reactivate an ES cell-like gene expression program in normal human cells and human cancer cells [35]. MYC overexpression has been shown to lead to cisplatin resistance in several *in vitro* models [36]–[39].

**Table 3:** Seventy-two Unique Genes Which Belong to ES cell-related Gene Sets (“ES Set without Proliferation Genes” and MYC/SOX2-Target Genes) *and* Were Upregulated in the Chemoresistant State at *P*<0.01.

Probeset	Gene	*t*-value^1^	Probeset	Gene	*t*-value
202840_at	TAF15	5.2	200901_s_at	M6PR	3.0
210350_x_at	ING1	4.2	210416_s_at	CHEK2	3.0
201247_at	SREBF2	3.9	37950_at	PREP	3.0
203391_at	FKBP2	3.9	209134_s_at	RPS6	3.0
218481_at	EXOSC5	3.8	208619_at	DDB1	3.0
210014_x_at	IDH3B	3.7	33132_at	CPSF1	3.0
208714_at	NDUFV1	3.7	217792_at	SNX5	3.0
204133_at	RRP9	3.6	202857_at	TMEM4	3.0
203103_s_at	PRPF19	3.6	211595_s_at	MRPS11	3.0
212563_at	BOP1	3.6	209147_s_at	PPAP2A	2.9
217874_at	SUCLG1	3.5	200812_at	CCT7	2.9
208676_s_at	PA2G4	3.5	201039_s_at	RAD23A	2.9
202339_at	SYMPK	3.4	209029_at	COPS7A	2.9
221809_at	RANBP10	3.4	201391_at	TRAP1	2.9
201487_at	CTSC	3.4	200658_s_at	PHB	2.9
211975_at	ZNF289	3.4	212357_at	KIAA0280	2.9
202072_at	HNRNPL	3.3	218405_at	ABT1	2.9
202649_x_at	RPS19	3.3	200637_s_at	PTPRF	2.9
209509_s_at	DPAGT1	3.3	218049_s_at	MRPL13	2.9
208907_s_at	MRPS18B	3.3	212191_x_at	RPL13	2.9
221669_s_at	ACAD8	3.3	200695_at	PPP2R1A	2.8
217940_s_at	FLJ10769	3.3	201834_at	PRKAB1	2.8
200980_s_at	PDHA1	3.3	218866_s_at	POLR3K	2.8
209196_at	WDR46	3.3	213175_s_at	SNRPB	2.8
210859_x_at	CLN3	3.2	210027_s_at	APEX1	2.8
202926_at	NAG	3.2	218670_at	PUS1	2.8
200824_at	GSTP1	3.2	209669_s_at	SERBP1	2.8
219979_s_at	C11orf73	3.2	212032_s_at	PTOV1	2.8
208101_s_at	URM1	3.1	200022_at	RPL18	2.8
208950_s_at	ALDH7A1	3.1	200819_s_at	RPS15	2.8
200874_s_at	NOL5A	3.1	204175_at	ZNF593	2.8
203039_s_at	NDUFS1	3.1	209224_s_at	NDUFA2	2.8
201732_s_at	CLCN3	3.1	209148_at	RXRB	2.8
202699_s_at	TMEM63A	3.1	216105_x_at	PPP2R4	2.8
200834_s_at	RPS21	3.1	217753_s_at	RPS26	2.8
201871_s_at	LOC51035	3.0	221475_s_at	RPL15	2.8

**Table 4:** Multivariable Regression Analyses of the 72-gene Predictive Index in 101 Separate (Non-rebiopsied) Gastric Cancer Patients.

	Time to progression^1^			Overall survival^1^			Radiographic response^2^		
	*P*	HR^3^	(95% CI^4^)	*P*	HR	(95% CI)	*P*	OR^5^	(95% CI)
**72-gene predictive index** 6	0.011	1.01	(1.001–1.009)^7^	0.034	1.004	(1.000–1.008)	0.036	1.008	(1.001–1.016)
**Poor performance status**									
(ECOG PS^8^ 2–3)^9^	0.048	2.31	(1.009–5.266)	0.049	2.240	(1.005–4.992)	0.452	1.847	(0.373–9.139)
**Age** 10	0.268	0.99	(0.965–1.010)	0.953	0.999	(0.976–1.023)	0.215	0.974	(0.934–1.015)
**Female**	0.100	1.57	(0.917–2.675)	0.156	1.462	(0.865–2.469)	0.564	1.370	(0.470–3.995)

1 Result of the Cox regression analysis performed for 101 patients.

2 Result of the ordinal logistic regression analysis performed only in 88 patients with measurable disease, using 3 categories of the dependent variable (PR, SD, and PD).

3 Hazard ratio.

4 Confidence interval.

5 Odds ratio.

6 Weighted linear combination of log signal values of 72-gene acquired resistance signature. The univariate *t*-statistics for comparing the acquired chemoresistant state with the pretreatment state were used as the weights.

7 Hazard ratio for each unit increase in 72-gene predictive index, which ranges from 1,783 to 2, 075 (*i.e.*, the highest predictive index (2,075) and median predictive index (1,945) are associated with hazard ratios of 4.3 ( = 1.005^292^) and 2.2 ( = 1.005^162^), respectively, compared with a hazard ratio of 1.0 with the lowest predictive index (1,783) of all 101 samples).

8 Eastern Cooperative Oncology Group Performance Status.

9 as compared with ECOG PS 0 or 1.

10 Hazard ratio for each year increase in age.

## Discussion

A major finding of this study is the identification of a gene signature that emerged in association with tumor resistance to CF therapy in patients who initially benefited from CF therapy. Prior genomic predictors for the chemotherapy response, which were developed using pretreatment tissue samples, have demonstrated a mixed performance [1], [2]. Here we demonstrate that the posttreatment samples collected at the time of acquired resistance, although difficult to obtain clinically, contain unique genomic information that can be used to predict the initial response to cytotoxic chemotherapy. No prior studies have explored acquired resistance using genome-wide analysis of clinical samples, although 2 prior studies evaluated the gene expression pattern in residual disease after the completion of neoadjuvant chemotherapy [40], [41]. Lee, *et al.* demonstrated that postchemotherapy tumor gene signatures outperforms baseline signatures and clinical predictors in predicting for pathological response and progression-free survival [42], although these investigators collected posttreatment breast tumors 3 weeks after chemotherapy, not at the time of progressive disease as in our study. Our data is consistent with the aforementioned study [42] that comparing postchemotherapy and prechemotherapy gene expression signatures might be a feasible approach to the identification of predictive signatures. Also, our data provides the first genomic evidence in clinical samples supporting a conventional model for the emergence of acquired resistance whereby resistance emerges through a selective, clonal outgrowth of small populations of pre-existing, chemoresistant tumor cells [3].

While the “*72-gene acquired resistance signature*” was developed mainly for potential clinical utility, it contains several overexpressed genes that have been shown to lead to chemoresistance. TRAP1 overexpression leads to 5-fluorouracil-, oxaliplatin- and irinotecan-resistant phenotypes in different neoplastic cells [29]. Silencing of hHR23A, a nucleotide excision repair (NER) enzyme, decreases the nuclear DRP1 level and cisplatin resistance in lung adenocarcinoma cells [31]. DDB1, which is also involved in NER, is overexpressed in cisplatin resistant cancer cell lines [32]. Elevated glutathione *S*-transferase P1 expression has been associated with resistance to cisplatin-based chemotherapy in several cancer cell lines [33], [34].

Our gene set comparison analyses demonstrate a significant overlap between the ES cell signatures and our chemotherapy resistance signatures. No prior studies have demonstrated the enrichment of ES cell signatures in clinical samples collected at the time of acquired resistance to cytotoxic chemotherapy. Accumulating evidence suggests an association between a stem cell phenotype and intrinsic chemoresistance [43]–[45]. Animal studies have suggested that the cell population exhibiting cancer stem cell characteristics is enriched in xenograft tumors following chemotherapy [46], [47]. While ES cell signatures may not perfectly reflect the phenotype of gastric cancer stem cells (which have not been defined yet), the enrichment of ES cell signatures in chemoresistant tumors may reflect the survival advantage of tumor cells expressing stem cell regulatory networks. This was validated by our finding that 72 genes shared by the acquired resistance and ES cell signatures were sufficient to predict the initial response to CF.

This study has identified a molecular signature for acquired resistance to CF therapy in gastric cancer patients. This signature is able to identify patients likely to have a short or longer term response to CF suggesting it reflects the molecular profile of chemoresistant clones and not non-specific drug effects. Genes contained within this signature, such as Akt/mTOR pathway genes, *TRAP1*, *RAD23A,* and *GSTP1*, may be potentially useful targets for treating tumors resistant to CF therapy. Future studies will be required to confirm these results and to determine whether our novel approach to develop an acquired resistance signature that predicts the therapeutic response of patients to specific chemotherapies is applicable to other types of cancer.

## Supporting Information

Figure S1 A modified Kegg pathway diagram for mTOR pathway in which genes belonging to 633-gene acquired resistance signature are shown in bold.
(TIF)

Table S1 Characteristics of Healthy Volunteers.
(DOC)

Table S2 Quality Control Parameters of Microarray Data for Patient Subsets Used for the Analyses.
(DOC)

Table S3 A Functional Category Significantly Enriched in Upregulated Genes in the Acquired Resistance (*P* for Feature Selection<0.01) according to the Ingenuity Pathway Analysis.
(DOC)

Table S4 Transcription Factor Target Gene Lists Which Have More Genes Differentially Expressed between Pretreatment and the Acquired Resistance State than Expected by Chance.
(DOC)

Table S5 LS *P* Values for Gene Comparison Analyses for Various Ranked Gene Lists of the BRB-ArrayTools Projects Using Published Stem Cell-related Gene Sets as User-defined Gene Sets.
(DOC)

Table S6 Gene Comparison Analyses for Acquired Resistance Signature^1^ Using Published Stem Cell Genesets as User-defined Genesets.
(DOC)
