# Supplementary figures and images for: A Gene Expression Signature of Acquired Chemoresistance to Cisplatin and Fluorouracil Combination Chemotherapy in Gastric Cancer Patients
Source: PLoS One. 2011 Feb 18;6(2):e16694. doi: 10.1371/journal.pone.0016694 (PMC3041770; doi:10.1371/journal.pone.0016694)

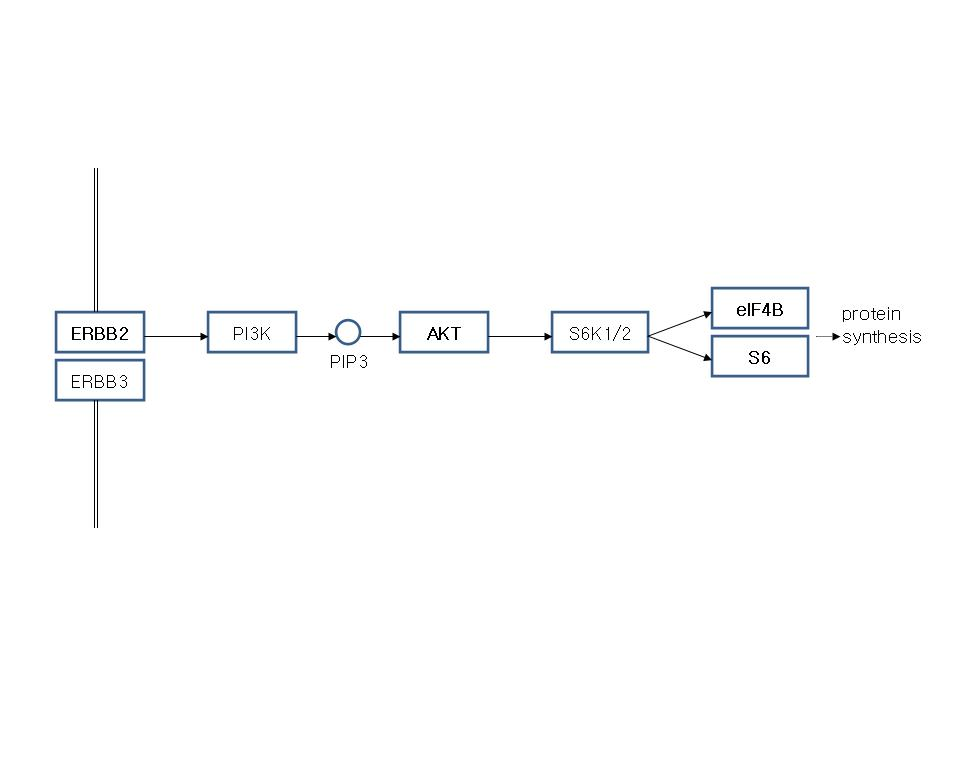

Supplement: Figure S1 — A modified Kegg pathway diagram for mTOR pathway in which genes belonging to 633-gene acquired resistance signature are shown in bold. (TIF) [file pone.0016694.s001.tif]
