# Supplementary material for: A Gene Expression Signature of Acquired Chemoresistance to Cisplatin and Fluorouracil Combination Chemotherapy in Gastric Cancer Patients
Source: PLoS One. 2011 Feb 18;6(2):e16694. doi: 10.1371/journal.pone.0016694 (PMC3041770; doi:10.1371/journal.pone.0016694)
Supplement: Table S1 — Characteristics of Healthy Volunteers. (DOC) [file pone.0016694.s002.doc]

| Table S1. Characteristics of Healthy Volunteers | |
| --- | --- |
|  |  |
| **Number of subjects** | 21 |
| **Age - years** |  |
| Median | 53 |
| Range | 40-66 |
| **Sex – no. (%)** |  |
| Male | 15 (71.4%) |
| Female | 6 (28.6%) |
