# Supplementary material for: A Gene Expression Signature of Acquired Chemoresistance to Cisplatin and Fluorouracil Combination Chemotherapy in Gastric Cancer Patients
Source: PLoS One. 2011 Feb 18;6(2):e16694. doi: 10.1371/journal.pone.0016694 (PMC3041770; doi:10.1371/journal.pone.0016694)
Supplement: Table S2 — Quality Control Parameters of Microarray Data for Patient Subsets Used for the Analyses. (DOC) [file pone.0016694.s003.doc]

| **Table S2.** Quality Control Parameters of Microarray Data for Patient Subsets Used for the Analyses | | | | |
| --- | --- | --- | --- | --- |
|  |  |  |  |  |
|  | Rebiopsied |  | Non-rebiopsied | Healthy |
|  | GC^1^ patients |  | GC patients | volunteers |
|  |  |  |  |  |
|  | Pre-CF | Post-CF |  |  |
|  |  |  |  |  |
|  | (N=22) | (N=22) | (N=101) | (N=21) |
|  |  |  |  |  |
| **% Present call** |  |  |  |  |
| Median | 57.9 | 60.3 | 58 | 61.6 |
| Interquartile range | 51.5-64.1 | 55.4-64.3 | 53.1-61.8 | 59.2-63.9 |
|  |  |  |  |  |
| **Scaling factor^2^** |  |  |  |  |
| Median | 0.7 | 0.5 | 0.7 | 0.6 |
| Interquartile range | 0.3-1.0 | 0.3-0.8 | 0.4-1.0 | 0.5-0.7 |
|  |  |  |  |  |
| **3'/5' GAPDH ratio** |  |  |  |  |
| Median | 1.7 | 1.5 | 1.7 | 1.1 |
| Interquartile range | 1.4-2.2 | 1.2-2.8 | 1.4-2.4 | 1.0-1.3 |
|  |  |  |  |  |
|  |  |  |  |  |
|  |  |  |  |  |
|  |  |  |  |  |
| ^1^Gastric cancer |  |  |  |  |
| ^2^Target signal, 100 |  |  |  |  |
