# Supplementary material for: A Gene Expression Signature of Acquired Chemoresistance to Cisplatin and Fluorouracil Combination Chemotherapy in Gastric Cancer Patients
Source: PLoS One. 2011 Feb 18;6(2):e16694. doi: 10.1371/journal.pone.0016694 (PMC3041770; doi:10.1371/journal.pone.0016694)
Supplement: Table S3 — A Functional Category Significantly Enriched in Upregulated Genes in the Acquired Resistance (P for Feature Selection<0.01) according to the Ingenuity Pathway Analysis. (DOC) [file pone.0016694.s004.doc]

| **Table S3.** A Functional Category Significantly Enriched in Upregulated Genes in the Acquired Resistance (*P* for Feature Selection<0.01) according to the Ingenuity Pathway Analysis | | |
| --- | --- | --- |
|  |  |  |
|  |  |  |
| Category | B-H^1^ *P* | Genes |
| Protein synthesis | 0.028 | ACY1, AKT1, ARIH2, CLN3, CLN6, CTSC, EIF3D, |
|  |  | EIF3E, EIF3F, EIF3H, EIF4B, FBXL6, HGS, MRPL11, |
|  |  | MRPL12, MRPL13, MRPL16, PREP, RAD23A, RPL13, |
|  |  | RPL14, RPL15, RPL18, RPL29, RPL3, RPL30, RPL4, |
|  |  | RPS11, RPS19, RPS6, RPS9, ST14, TUFM |
|  |  |  |
|  |  |  |
| ^1^Benjamini-Hochberg |  |  |
