# Supplementary material for: A Gene Expression Signature of Acquired Chemoresistance to Cisplatin and Fluorouracil Combination Chemotherapy in Gastric Cancer Patients
Source: PLoS One. 2011 Feb 18;6(2):e16694. doi: 10.1371/journal.pone.0016694 (PMC3041770; doi:10.1371/journal.pone.0016694)
Supplement: Table S4 — Transcription Factor Target Gene Lists Which Have More Genes Differentially Expressed between Pretreatment and the Acquired Resistance State than Expected by Chance. (DOC) [file pone.0016694.s005.doc]

| Table S4. Transcription Factor Target Gene Lists Which Have More Genes Differentially Expressed between Pretreatment and the Acquired Resistance State than Expected by Chance | | |
| --- | --- | --- |
|  |  |  |
| Gene set | *No* genes | LS *P* value |
|  |  |  |
| ATF1_T00968 | 151 | < 10^-5^ |
| MYC_T00140 | 1,190 | < 10^-5^ |
| CEBPA_T00105 | 258 | < 10^-5^ |
| CREB1_T00163 | 342 | < 10^-5^ |
| E2F-1_T01542 | 619 | < 10^-5^ |
| E2F-2_T01544 | 176 | < 10^-5^ |
| E2F-4_T01546 | 406 | < 10^-5^ |
| EGR1_T00241 | 185 | < 10^-5^ |
| ESR1_T00261 | 203 | < 10^-5^ |
| ETS1_T00112 | 275 | < 10^-5^ |
| ETV4_T00685 | 137 | < 10^-5^ |
| HIF1A_T01609 | 123 | < 10^-5^ |
| JUN_T00029 | 360 | < 10^-5^ |
| MYB_T00137 | 301 | < 10^-5^ |
| NFIC_T00176 | 182 | < 10^-5^ |
| NFKB1_T00591 | 347 | < 10^-5^ |
| POU2F1_T00641 | 177 | < 10^-5^ |
| PPARG_T02736 | 124 | < 10^-5^ |
| RARA_T00719 | 212 | < 10^-5^ |
| RELA_T00594 | 146 | < 10^-5^ |
| SP1_T00759 | 566 | < 10^-5^ |
| SP3_T02338 | 171 | < 10^-5^ |
| SPI1_T02068 | 163 | < 10^-5^ |
| TFAP2A_T00035 | 529 | < 10^-5^ |
| TP53_T00671 | 469 | < 10^-5^ |
| USF1_T00874 | 184 | < 10^-5^ |
| USF2_T00878 | 140 | < 10^-5^ |
