# Supplementary material for: A Gene Expression Signature of Acquired Chemoresistance to Cisplatin and Fluorouracil Combination Chemotherapy in Gastric Cancer Patients
Source: PLoS One. 2011 Feb 18;6(2):e16694. doi: 10.1371/journal.pone.0016694 (PMC3041770; doi:10.1371/journal.pone.0016694)
Supplement: Table S5 — LS P Values for Gene Comparison Analyses for Various Ranked Gene Lists of the BRB-ArrayTools Projects Using Published Stem Cell-related Gene Sets as User-defined Gene Sets. (DOC) [file pone.0016694.s006.doc]

| Table S5. LS P Values for Gene Comparison Analyses for Various Ranked Gene Lists of the BRB-ArrayTools Projects Using Published Stem Cell-related Gene Sets as User-defined Gene Sets | | | |
| --- | --- | --- | --- |
|  |  |  |  |
|  |  |  |  |
| User-defined gene set | Ranked gene list | |  |
|  |  |  |  |
|  | Gastric cancer | Differentation | Intrinsic resistance |
|  | signature | signature | signature |
|  |  |  |  |
|  | (101 GC^1^ vs 21 normal) | (Diffuse vs intestinal) | (TTP of 101 GC) |
|  |  |  |  |
| ES expression set^2^ | NS^4^ | NS | <10^-5^ |
| ES set without proliferation genes^3^ | NS | NS | 6.0x10^-5^ |
| MYC target genes | NS | NS | <10^-5^ |
| SOX2 target genes | NS | NS | NS |
| OCT4 target genes | NS | 7.7x10^-4^ | NS |
| NANOG target genes | NS | 4.2x10^-3^ | NS |
| SUZ12 target genes | NS | NS | NS |
| EED target genes | NS | NS | NS |
| H3K27 target genes | NS | NS | NS |
|  |  |  |  |
|  |  |  |  |
|  |  |  |  |
| ^1^gastric cancer patients |  |  |  |
| ^2^Genes over-expressed in ES cells in 5 or more profiling studies^14^ | | |  |
| ^3^Amended gene set in which genes in the “proliferation” Gene Ontology and the proliferation cluster of breast cancer^13,15^ were excluded from ES expression set | | | |
| ^4^Not significant (P> 0.005) |  |  |  |
