# Supplementary material for: A Gene Expression Signature of Acquired Chemoresistance to Cisplatin and Fluorouracil Combination Chemotherapy in Gastric Cancer Patients
Source: PLoS One. 2011 Feb 18;6(2):e16694. doi: 10.1371/journal.pone.0016694 (PMC3041770; doi:10.1371/journal.pone.0016694)
Supplement: Table S6 — Gene Comparison Analyses for Acquired Resistance Signature1 Using Published Stem Cell Genesets as User-defined Genesets. (DOC) [file pone.0016694.s007.doc]

| Table S6. Gene Comparison Analyses for Acquired Resistance Signature^1^ Using Published Stem Cell Genesets as User-defined Genesets | | | |
| --- | --- | --- | --- |
|  |  |  |  |
|  |  |  |  |
| User-defined gene set |  |  | LS *P* value |
|  |  |  |  |
| Name | *No* Genes | Reference |  |
|  |  |  |  |
| ES expression set^2^ | 336 | 13 | 3.0x10^-3^ |
| ES set without proliferation genes^3^ | 288 | 13,15 | 4.0x10^-3^ |
| MYC target genes | 749 | 16 | 1.0x10^-5^ |
| SOX2 target genes | 636 | 17 | 4.3x10^-4^ |
| OCT4 target genes | 290 | 17 | NS^4^ |
| NANOG target genes | 839 | 17 | NS |
| SUZ12 target genes | 721 | 18 | NS |
| EED target genes | 731 | 18 | NS |
| H3K27 target genes | 759 | 18 | NS |
|  |  |  |  |
|  |  |  |  |
|  |  |  |  |
|  |  |  |  |
| ^1^A ranked gene list in which a P value was computed for each gene for the differential expression between the pretreatment- and chemoresistant-states of 22 rebiopsied responders | | | |
| ^2^Genes over-expressed in ES cells in 5 or more profiling studies^14^ | | |  |
| ^3^Amended gene set in which genes in the “proliferation” Gene Ontology and the proliferation cluster of breast cancer^13,15^ were excluded from ES expression set | | | |
| ^4^Not significant (P> 0.005) |  |  |  |
